# Military versus Medical Titles

**Published:** 1918-11-16

**Authors:** 


### MILITARY VERSUS MEDICAL TITLES.

THE post-war period will bring problems of its own, and the medical profession will share in the general experience. In addition the profession will have its special difficulties, and some of these are likely to prove considerable. There will be others of lesser order, and among these can be seen a question of names or titles. Just now many practitioners, known heretofore by a purely professional title, bear military rank and are addressed in accordance with military standards. An occasional purist may have insisted on the restriction of this usage to the period of purely military relation, but for the most part the "colonel" or the "major," or whatever it may be, has found recognition even in civilian affairs. The termination of temporary commissions will restore numerous officers of the R.A.M.C. to their former activities, and it may be supposed that in many instances permission will be granted to such officers to use the titular index appropriate to the military rank they have occupied. The question will, therefore, inevitably arise whether this permission is to be acted on in such a fashion as to establish a general custom. Is it, in other words, to be the rule and practice that doctors retiring from the Army shall revert in name as well as in substance to their civilian status, or, on the other hand, shall these gentlemen be addressed by the military titles to which they have a technical right? In essence the matter is a very small one, but it contains possibilities of irritation and friction, and it is desirable that it be settled in accordance with a general rule.

There is, it may be admitted, something to be said for the continued use of the military titles. The bearers of them have a right to be proud of their association with the fighting men by whom the war has been won, and the Medical Services of the Navy and Army can claim traditions and achievements both many and distinguished. If some members have rested safely at home others have been in the thick of the fight, and all have taken duty in the positions authoritatively commanded. Hence, considered as a body of men who have served their country more or less actively in the war, the civilian doctors may reasonably claim any appropriate recognition, and may well desire to preserve the memory of an association of which they have a right to be proud. Yet, on the whole, we believe they will be well advised to discourage the use of titles signifying military rank. In the Army these titles are necessary both to indicate status and to secure discipline. It is for these purposes they are accepted by medical practitioners, and not to suggest that the bearer's profession needs some decorative addition. When a more or less artificial status and discipline are no longer necessary the natural thing will be for the man to resume the title which is appropriate to the profession of his choice, and thus to indicate that his ambition is medicine and not the art of war. Each of the two professions has a certain position in the social order, and each is deserving of recognition and honour. But the two groups differ in aim and differ in method, and the titles which are perfectly seemly in one setting are apt to become a little ridiculous when transferred to other and different surroundings. The way to avoid such a result is for every man to abstain from labels having the form of accuracy which depends solely on a verbal defence. Perhaps a note in the direction here indicated may come from the events of the day and hour. Realities are asserting themselves, and much fair-seeming sham is withering in the flame. In no department of life is pretence and unreality more contemptible than in medicine, and it is well to avoid even the appearance of evil.

